# Formulation of objective indices to quantify machine failure risk analysis for interruptions in radiotherapy

**DOI:** 10.1002/acm2.13126

**Published:** 2020-12-16

**Authors:** Daisuke Kawahara, Hisashi Nakano, Akito Saito, Yusuke Ochi, Yasushi Nagata

**Affiliations:** ^1^ Department of Radiation Oncology Institute of Biomedical & Health Sciences Hiroshima University Hiroshima Japan; ^2^ Department of Radiation Oncology Niigata University Medical and Dental Hospital Niigata Japan; ^3^ Radiation Therapy Section Department of Clinical Support Hiroshima University Hospital Hiroshima Japan; ^4^ Hiroshima High‐Precision Radiotherapy Cancer Center Hiroshima Japan

**Keywords:** biological dose calculation, downtime, machine failure

## Abstract

**Objectives:**

To evaluate the effect of interruption in radiotherapy due to machine failure in patients and medical institutions using machine failure risk analysis (MFRA).

**Material and methods:**

The risk of machine failure during treatment is assigned to three scores (biological effect, *B*; occurrence, *O*; and cost of labor and repair parts, *C*) for each type of machine failure. The biological patient risk (BPR) and the economic institution risk (EIR) are calculated as the product of B and O (B×O) and C and O (C×O), respectively. The MFRA is performed in two linear accelerators (linacs).

**Result:**

The multileaf collimator (MLC) fault has the highest BPR and second highest EIR. In particular, TrueBeam has a higher BPR and EIR for MLC failures. The total EIR in TrueBeam was significantly higher than that in Clinac iX. The minor interlock had the second highest BPR, whereas a smaller EIR. Meanwhile, the EIR for the LaserGuard fault was the highest, and that for the monitor chamber fault was the second highest. These machine failures occurred in TrueBeam. The BPR and EIR should be evaluated for each linac. Further, the sensitivity of the BPR, it decreased with higher $T_{1/2}$ and α/β values. No relative difference is observed in the BPR for each machine failure when $T_{1/2}$ and *α*/*β* were varied.

**Conclusion:**

The risk faced by patients and institutions in machine failure may be reduced using MFRA.

**Advances in knowledge:**

For clinical radiotherapy, interruption can occur from unscheduled downtime with machine failures. Interruption causes sublethal damage repair. The current study evaluated the effect of interruption in radiotherapy owing to machine failure on patients and medical institutions using a new method, that is, machine failure risk analysis.

## 1 INTRODUCTION

A linear accelerator (linac) that facilitates the treatment of cancer comprises complex hardware and software. Development of the linac has progressed steadily, and the reliability and consistency of its operations have improved remarkably. However, linacs still occasionally suffer occasionally from system dysfunction and failure. Component dysfunction or failure calls for service engineering and immediate on‐site repair, resulting in the disruption of clinical services and unscheduled machine downtime (DT) ^1^, ^2^. This presents a strain on the patients and involves a cost for the institution. Linac interlocks prevent grave failures by ensuring that the operation of the system is discontinued when the operating parameters exceed the specified limits of the system. However, such interlocks can also cause DT, thereby affecting clinical operations.

Sublethal damage repair (SLDR) is induced in patients for several minutes or hours after irradiation for patients, and it causes decreased cell killing in a certain time fraction. In clinical treatments, an interruption can occur from (1) unscheduled DT with machine failures, (2) increasing the interval between treatment beams through couch rotations with the non‐coplanar beams, and (3) increasing the interval between multiple beams. Shibamoto et al. evaluated the dose difference with and without interruptions in in‐vivo experiments.^3^ Cell survival increased by 13% in mammary cell carcinoma, EMT6, and by 18% in mouse head and neck squamous cell carcinoma, SCCVII, with a 5‐min interval ^4^, ^5^. The effect on cell survival with multiple interruptions, such as intensity‐modulated radiation therapy, may be less than those of the same dose without interruptions. Moreover, the effect of cell survival with SLDR appears to almost plateau after several hours of interruption.^6^ Brenner et al. suggested a linear–quadratic (LQ) model with the Lea–Catcheside time factor to analyze cell survival considering SLDR during irradiation at the cell population level. ^7^

In this study, the biological effect of the duration of interruption caused by machine failure was determined using the LQ model with the Lea–Catcheside time factor of a single interruption in one fraction, and a risk analysis with SLDR by machine failures was emphasized. For an institution, the cost associated with machine DT is a significant factor to consider as the costs associated with health systems must be economically sustainable.^8^, ^9^ Hence, machine failure presents a high risk for medical institutions. The current study proposes a new machine failure risk analysis (MFRA) method that involves calculating the cost of replacement of linac parts and the biological effect of DT on patients. Furthermore, the machine failure risk faced by patients and institutions during treatment for each machine failure was analyzed for two linacs.

## 2 MATERIALS AND METHODS

Two linacs (Clinac iX; Varian Medical Systems, Palo Alto, USA) and TrueBeam (Varian Medical Systems, Palo Alto, USA) were used for this study. Clinac iX was introduced in 2009, while TrueBeam was introduced in 2013.

### 2.A Classification of machine failure

Table 1 lists the total number of unplanned intra‐fraction machine failures on a treatment session from April 2015 to April 2018. The total number of unplanned intra‐fraction machine failures was 60. Based on the 60 failure modes identified, the machine failures were broadly classified into the following categories: multileaf collimator (MLC), potentiometer, radio frequency (RF) driver, minor interlock, water temperature and quantity, monitor chamber and LaserGuard. The failure associated with the MLC includes failures associated with the motor, communication, leaf and carriage positioning, and power supply. If the water temperature increases, low water and gas pressures or other problems cause an interruption during irradiation to decrease the temperature. Failures associated with the potentiometer and RF driver involve an active interlock, which turns the beam off during irradiation. These machine failures must be addressed by replacing the parts. The TrueBeam linac has a collision detection system called LaserGuard, which comprises an infrared laser. LaserGuard is used to replace the parts when the interlock associated with the collision cannot be released. The minor interlocks alert the operator to the existence of conditions that affect machine operation, such as filament time delay, calibration cycle timeout, and excess dose rate. This interlock does not require a significant amount of time to release the interlock, part replacement or a system restart. It is released by re‐mode up, username and password input.

**TABLE 1:** Total number of unplanned intra‐fraction machine failures and downtime (DT; min).

Machine failure	0 < DT ≤ 10	10 < DT ≤ 20	20 < DT ≤ 30	30 < DT ≤ 40	50 < DT ≤ 60	80 ≤ DT ≤ 90	DT ≥ 100
MLC	4 (3,1)	6 (2,4)	6 (4,2)	0	1 (1,0)	0	2 (1,1)
Potentiometer	4 (0,4)	0	0	1 (1,0)	0	0	1 (1,0)
RF driver	0	0	1 (0,1)	1 (0,1)	0	0	2
Minor interlock	18 (7,11)	0	2 (0,2)	1	0	0	0
Water	2 (2,0)	0	0	3 (3,0)	0	0	1 (1,0)
Cable	0	0	1 (0,1)	0	0	0	1 (0,1)
Monitor chamber	1 (1,0)	0	0	0	0	0	0
LaserGuard	0	0	0	0	0	0	1 (1,0)

### 2.B Machine failure risk analysis

MFRA is performed to calculate the risk faced by patients and institutions by evaluating the cost of repair and biological effects when DT occurs. The risk of machine failure during treatment can be assigned to the following three scores: biological effect, *B*; occurrence, *O*; and labor and repair part, *C*. The current study focused on machine failure, without swapping clinical treatment plans among beam‐matched linacs, and no rescheduling time is available after treatment of all patients.

### 2.C Biological effects in treatment

In this study, we focused on the biological effects of an unplanned intra‐fraction break in a treatment session caused by a machine malfunction, as shown in Table 1. We assume that, except for this delay, the total dose for the session was delivered as planned without swapping with a clinically beam‐matched linac and that no rescheduling time was available for a patient to continue treatment after the daily treatment of all patients. Moreover, in the current study, it was assumed that biological effects follow the LQ model, which provides a simple relationship between cell survival and delivered dose.^10^, ^11^ More importantly, the standard LQ formalism, as applied to time–dose relationships, is not merely a truncated power series in dose. The key feature is a specific mechanistically based functional form for the protraction factor (*G*), which considers dose protraction or fractionation. This factor was derived by Lea and Catcheside.^12^, ^13^ Brenner et al. applied *G* to the biological dose calculation and calculated the survival fraction in the case of two acute dose fractions,$D_{1}$ and $D_{2}$, separated by the DT using the LQ formalism that was incorporated as well as the protraction factor.^7^ The biological effect with interruption depends on the DT and dose per fraction (DPF). In the current study, it was assumed that the interruption occurred during one‐half of the irradiation. The survival fraction with interruption ($SF^{\mathrm{with}}$) is calculated as follows:

(1) $$SF^{\mathrm{with}}=\exp\left[‐(\alpha D_{1}+\alpha D_{2}+\beta D_{1}^{2}+\beta D_{2}^{2}+2\beta D_{1}D_{2}e^{‐\lambda T})\right].$$

**FIG. 1:**
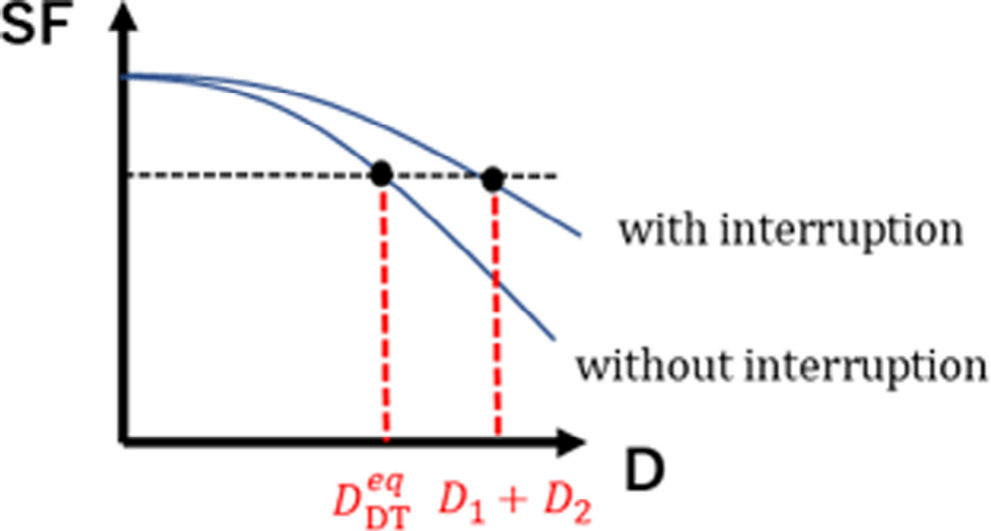
Correlation between survival fraction and DPF with and without interruption.

Here, λ is the repair rate for double‐strand breaks, equal to 2/$T_{1/2}$, where $T_{1/2}$ is the repair half‐time. Typical values of α/β are used for early responding tissues, as listed in Table 2.7 These were also used in the simulation by Brenner et al.^14^ The survival fraction without interruption equivalent to the survival fraction with interruption is denoted as $SF^{w/o}$. . The equivalent DPF is defined as $D_{\mathrm{DT}}^{\mathrm{eq}}$ when $SF^{\mathrm{with}}=SF^{w/o}$, as shown in Fig. 1. $SF^{w/o}$ is calculated as follows:

(2) $$SF^{w/o}=exp\left[‐\left(\alpha D_{\mathrm{DT}}^{\mathrm{eq}}+\beta D_{\mathrm{DT}}^{\mathrm{eq}}{\;}^{2}\right).\right]$$

**TABLE 2:** Parameters used in the calculation.

Parameters	Values
α (Gy^‐1^)	0.12
β (Gy^‐2^)	0.0137
T_1/2_ (h)	0.35

The $D_{\mathrm{DT}}^{\mathrm{eq}}$ value can be calculated from Eqs. (1) and (2) as follows:

(3) $$D_{\mathrm{DT}}^{\mathrm{eq}}=‐\frac{\alpha }{2\beta }+\sqrt{{\left(\frac{\alpha }{2\beta }\right)}^{2}+\frac{\alpha }{\beta }\left(D_{1}+D_{2}\right)+\left(D_{1}^{2}+D_{2}^{2}\right)+2D_{1}D_{2}e^{‐\lambda \cdot DT}}.$$

Additionally, the biological effect of DT for each DPF is denoted as $B_{DT,DPF}$. To calculate $B_{DT,DPF}$, the maximum DT was used when the machine failures were classified every 10 min in the range of 0–100 min, as shown in Table 1. For example, when one machine failure occurred with a DT of 0–10 min, then 10 min was used as the DT value in the calculation of $B_{DT,DPF}$. Additionally, a DT exceeding 100 min was assigned 100 min. Subsequently, $B_{DT,DPF}$ can be obtained using:

(4) $$B_{DT,DPF}=\frac{D_{DT,DPF}^{\mathrm{eq}}‐D_{DT=0,DPF}^{\mathrm{eq}}}{D_{DT=0,DPF}^{\mathrm{eq}}}\times 100.$$

### 2.D Occurrence

Occurrence in MFRA is defined as the probability of machine failure for the DPF of each patient when DT occurs ($o_{DT,DPF,MF}$). Information regarding machine failures and DT is shown in Table 1. In practice, the occurrence is calculated as follows:

(5) $$o_{DT,DPF,MF}=\frac{N_{DT,DPF,MF}}{\mathrm{treatmentperiod}}.$$

where $N_{DT,DPF,MF}$ is the number of machine failures for the DPF of each patient when a machine failure with a DT occurs. The evaluation period used was 36 months, which was equivalent to the analysis period.

### 2.E Cost of labor and repair parts

The cost of labor is directly related to the payment of the treatment staff. In the current study, it was assumed that a backup machine would not be used. The cost was analyzed based on machine failures that occurred during an unplanned intra‐fraction break in a treatment session, as shown in Table 1. Thus, this study focused on the labor cost and repair parts for an unplanned intra‐fraction break in a treatment session. The labor cost incurred by two therapists and nurses in treating a patient when machine failures did not occur was considered. Meanwhile, various cases pertaining to the labor cost of an engineer exist. Some hospitals hire engineers or establish a maintenance contracts with vendors. Therefore, in the current study, the labor costs of two therapists and nurses were considered. Moreover, cost of a physicist was not included in the economic institution risk (EIR) analysis because flexible working hours are applied to most physicists and their salary is not paid hourly. The cost of radiotherapy has been categorized based on high and low‐income countries by Van Dyk et al.^15^ Using the monthly salary and working time specified by Van Dyk et al., the total salary of two therapists and nurses per minute S was calculated as follows:

(6) $$S=\frac{2\times \left[Therapist\;Salary\right]+1\times \left[Nurse\;Salary\right]}{working\;time\left(\min\right)}.$$

*S* was approximately $1.06/min. In addition, the cost of labor for the event i of machine failure for each DT and DPF $c_{i}^{\mathrm{labor}}$was calculated using.

(7) $$c_{i}^{\mathrm{labor}}=S\times t_{i}.$$

where $t_{i}$ is the maximum DT when machine failures are divided into intervals of 10 min in the range of 0–100 min, as shown in Table 1. For example, when one machine failure occurs with a DT of 0–10 min, the DT value used in the calculation of $c_{i}^{\mathrm{labor}}$ is 10 min. Further, a DT exceeding 100 min was assigned 100 min. The cost of repair using replacement parts of each machine failure for event *i* of the machine failure for each DT and DPF is denoted as $c_{i}^{\mathrm{parts}}$. Thus, the total cost including the costs of labor and repair using replacement parts for each event *i* of machine failure $c_{i}^{\mathrm{total}}$ is calculated as follows.

(8) $$c_{i}^{\mathrm{total}}=c_{i}^{\mathrm{labor}}+c_{i}^{\mathrm{parts}}.$$

### 2.F Biological patient risk (BPR) and economic institution risk (EIR)

The biological effect of machine failure on patients is defined as the biological patient risk (BPR) based on the ratio of the number of patients for each DPF, denoted as $BPR_{DT,DPF,MF}$. It can be calculated as follows:

(9) $$BPR_{DT,DPF,MF}=B_{DT,DPF,MF}\times o_{DT,DPF,MF}$$

where $BPR_{DT,DPF,MF}$ is the $BPR_{DT,DPF}$ for each machine failure. $BPR_{\mathrm{MF}}$ is calculated using the total DT at each DPF for each machine failure.

(10) $$BPR_{\mathrm{MF}}=\sum_{DT,DPF}BPR_{DT,DPF,MF}$$

For economic analysis, the EIR for each machine failure ($EIR_{\mathrm{MF}}$) is defined as.

(11) $$EIR_{\mathrm{MF}}=\frac{\sum_{i=1}^{DT,DPF}c_{i}^{\mathrm{total}}}{\sum_{i=1}^{DT,DPF,MF}c_{i}^{\mathrm{total}}}$$

An example of BPR calculation for water temperature and quantity faults is presented here. This fault occurred three times with DT of 0–10 min and B=3.3%, and once with DT of 30–40 min and B=5.4% in 2 Gy/fr patients. The calculation is given by:

(12) $$BPR_{2Gy/fr}=3.3\left(\%\right)\times \frac{2\left(\mathrm{time}\right)}{36\left(\mathrm{month}\right)}+5.4\left(\%\right)\times \frac{1\left(\mathrm{time}\right)}{36\left(\mathrm{month}\right)}=0.33.$$

Moreover, the water temperature and quantity faults occurred once with a DT of 30–40 min and B=8.0%, and twice with a DT exceeding 100 min and B=8.2% in 3 Gy/fr patients. This was calculated as follows:

(13) $$BPR_{3Gy/fr}=8.0\left(\%\right)\times \frac{2\left(\mathrm{time}\right)}{36\left(\mathrm{month}\right)}+8.2\left(\%\right)\times \frac{1\left(\mathrm{time}\right)}{36\left(\mathrm{month}\right)}=0.67.$$

For the other DPF, the faults of water temperature and quantity did not occur. The total $BPR_{\mathrm{MF}}$ with 2–20 Gy for these faults is calculated using:

(14) $$BPR_{\mathrm{MF}}=0.33+0.67=1.00.$$

### 2.G Sensitivity of BPR

Leeuwen et al. reported large variations in their published values for the LQ parameters *α*/*β* between different tumor types.^16^ Additionally, they reported that variations appeared in a study of the same tumor type. This is known as study heterogeneity, which occurs in studies where a value is estimated that is only valid for a specific method and the patient cohort of that particular study. Hence, the sensitivity of the BPR was investigated for variations of *α*/*β* and $T_{1/2}$, which are the parameters of the LQ model with the Lea‐Catcheside time factor, respectively. The *α*/*β* was 2 Gy for typical late‐responding normal tissues and 10 Gy for typical early‐responding normal tissues and tumors. In addition to the typical values for early‐responding tissues shown by Brenner et al.,^7^ $T_{1/2}$ was 0.2 and 0.5 hr.

### 2.H Sensitivity of EIR

The sensitivity of $EIR_{\mathrm{MF}}$ to the variation in labor cost was investigated. The labor cost was eliminated by assuming that a backup machine can be used, and that all patients with machine failures can be transferred to the backup machine without overtime. The EIR for each machine failure without a backup machine is defined by Eq. 12. The EIR for each machine failure with a backup machine ($EIR_{\mathrm{MF}}^{\mathrm{BA}}$) is defined as the ratio of the cost of repair parts to the total cost:

(15) $$EIR_{\mathrm{MF}}^{\mathrm{BA}}=\frac{c_{i}^{\mathrm{parts}}}{\sum_{i=1}^{DT,DPF,MF}c_{i}^{\mathrm{total}}}.$$

## 3 RESULTS

### 3.A Biological effects with downtime

**FIG. 2:**
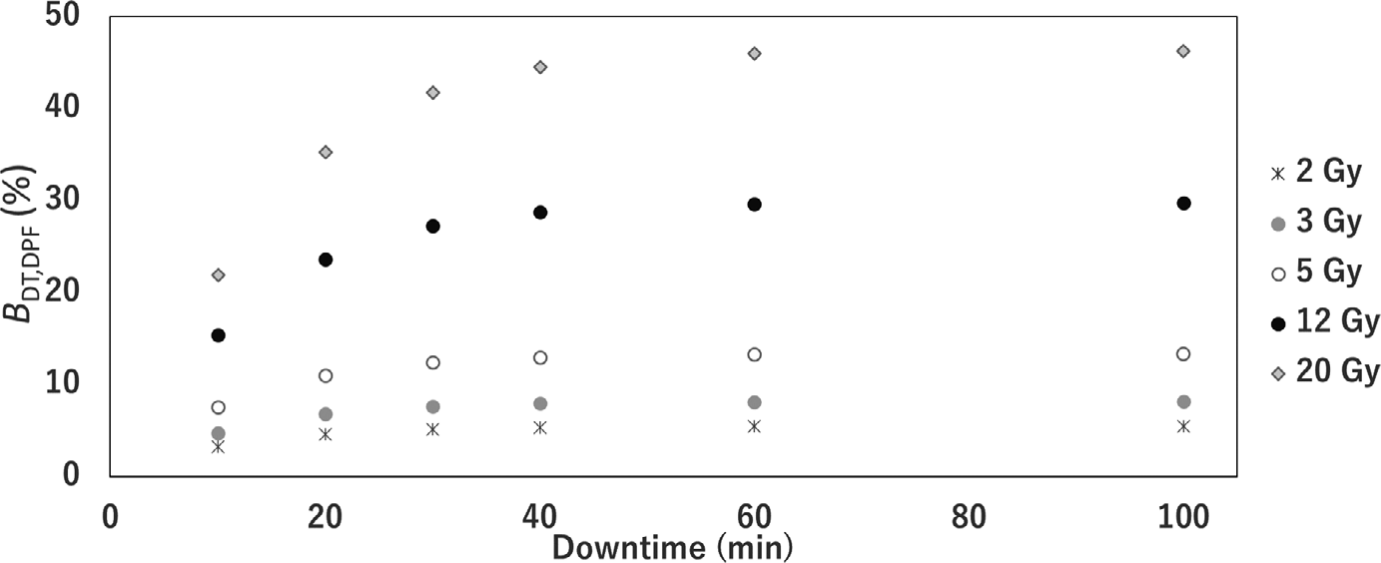
$B_{DT,DPF}$ with a downtime of 0–100 min at doses of 2–20 Gy with cell parameters shown in Table 2 (α = 0.12 Gy^‐1^, β = 0.0137 Gy^‐1^, and T_1/2_ *= 0.35*).

Figure 2 shows the $B_{DT,DPF}$ vs. DTs of 0–100 min for the doses—2, 3, 5, 10, and 20 Gy with the cell parameters shown in Table 2. *B* increased with higher doses and longer DT. In particular, the rate of increase of $B_{DT,DPF}$ until the DT of 40 min was higher for increased doses.

### 3.B BPR and EIR for both linear accelerators

**FIG. 3:**
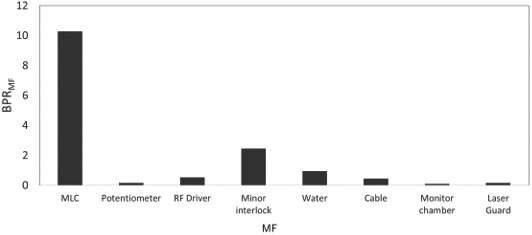
BPR_MF_ for machine failures caused by MLC, potentiometer, RF driver, minor interlock, water temperature and quantity, cable, monitor chamber, and laser guard in TrueBeam and Clinac iX.

**FIG. 4:**
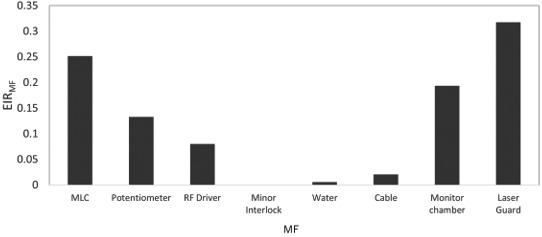
EIR_MF_ for machine failures caused by MLC, potentiometer, RF driver, minor interlock, water temperature and quantity, cable, monitor chamber, and laser guard in TrueBeam and Clinac iX.

Figures 3 and 4 show the BPR_MF_ and EIR_MF_ with both linacs. The MLC fault had the highest BPR_MF_ and the second highest EIR_MF_. The EIR_MF_ for the LaserGuard fault was the highest. Meanwhile, the minor interlock had the second highest BPR_MF_, whereas it had the smallest EIR_MF_. The EIR_MF_ for the monitor chamber fault was the second highest, while the BPR_MF_ was the smallest. Additionally, the BPR_MF_ and EIR_M_ for the water temperature and quantity faults were higher and smaller, respectively than those of machine failures caused by the potentiometer, RF driver and cable.

### 3.C BPR_MF_ and EIR_MF_ for each machine

**FIG. 5:**
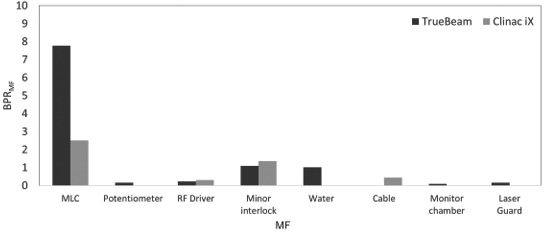
BPR_MF_ for machine failures caused by MLC, potentiometer, RF driver, minor interlock, water temperature and quantity, cable, monitor chamber, and LaserGuard in TrueBeam and Clinac iX.

**FIG. 6:**
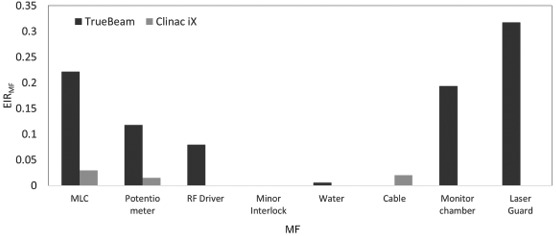
EIR_MF_ for machine failures caused by MLC, potentiometer, RF driver, minor interlock, water temperature and quantity, cable, monitor chamber, and LaserGuard in TrueBeam and Clinac iX.

Figures 5 and 6 show the BPR_MF_ and EIR_MF_ in both TrueBeam and Clinac iX. TrueBeam had the highest BPR_MF_ for MLC failures, whereas, Clinac iX had the highest BPR_MF_ for minor interlock failure. The total EIR_MF_ in TrueBeam was significantly higher than that in Clinac iX. The EIR_MF_ was the highest for the LaserGuard fault in TrueBeam. Although Clinac iX had the highest EIR_MF_ for MLC failures, the value was smaller than that of TrueBeam. The BPR_MF_ from a minor interlock fault, _F_ was the second highest; whereas, its EIR_MF_ was smaller for both linacs. Although the BPR_MF_ for RF driver failure in TrueBeam was larger than that in Clinac iX, the EIR_MF_ for RF driver failure in Clinac iX was larger than that in TrueBeam. Although the minor interlock fault in Clinac iX had a higher BPR_MF_, it had a smaller EIR_MF_ in TrueBeam.

### 3.D Sensitivity analysis of BPR_MF_

**FIG. 7:**
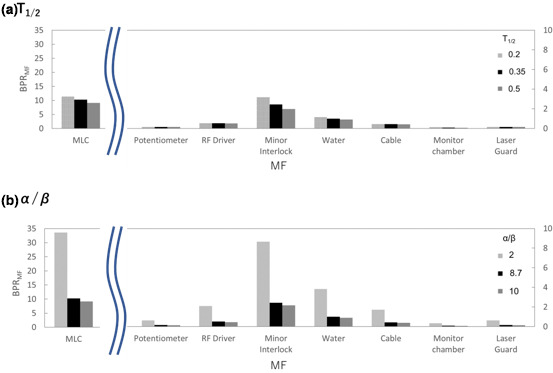
BPR_MF_ for machine failures caused by MLC, potentiometer, RF driver, minor interlock, water temperature and quantity, cable, monitor chamber, and LaserGuard in TrueBeam and Clinac iX with (a) $T_{1/2}$ of 0.2–0.5 and α/β of 10 Gy, and (b) $T_{1/2}$ of 0.35 and α/β of 2–10 Gy.

Figure 7 shows the variations in BPR_MF_ for the machine failures caused by the MLC, potentiometer, RF driver, minor interlock, water temperature and quantity, cable, monitor chamber and LaserGuard. The BPR_MF_ decreased with higher $T_{1/2}$ and α/β values.

### 3.E Sensitivity of EIR

**FIG. 8:**
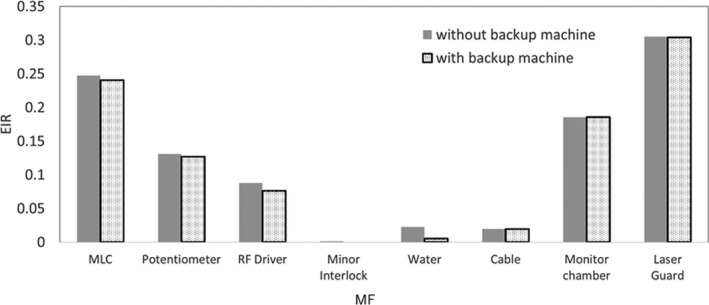
EIR_MF_ for machine failures caused by MLC, potentiometer, RF driver, minor interlock, water temperature and quantity, cable, monitor chamber, and LaserGuard with and without backup machine.

Figure 8 shows the variations in EIR_MF_ for the machine failures caused by the MLC, potentiometer, RF driver, minor interlock, water temperature and quantity, cable, monitor chamber and LaserGuard with and without a backup machine. The EIR_MF_ for the MLC, potentiometer, and RF driver faults decreased slightly when the backup machine was used. In particular, the EIR_MF_ for the water temperature and quantity faults reduced by more than one half when the backup machine was used. Meanwhile, the EIR_MF_ values of the cable, monitor chamber, and LaserGuard with and without a backup machine almost did not differ.

## 4 DISCUSSION

The B_DT,DPF_ increased with higher DPF and DT, as shown in Fig. 2. In clinical practice, the treatment technique and DPF differ for each patient. The difference between the DT and DPF is considered in the BPR for machine failures. In this study, the effects of multiple treatments on BPR were not considered. We would expect the BPR to be reduced for multi‐fraction treatments as opposed to single‐fraction treatments. The current study focused on machine failure with a high BPR. Although the DT caused by the minor interlock is not very long, the highest BPR would depend on the probability of occurrence of the minor interlock fault. The total BPR for the MLC was the highest, and it was significantly higher in TrueBeam. The time elapsed since linac installation may affect the number of machine failures. Therefore, the BPR should be evaluated for each linac. Wroe et al. analyzed machine failures in the UK, Nigeria, and Botswana.^17^ They reported that the number of machine failures in MLCs was larger immediately after a linac is introduced. Machine failures 3–6 a after the introduction of TrueBeam and 7–10 a after the introduction of Clinac iX were analyzed. It was discovered that the frequency of machine failures was larger in TrueBeam. However, the sample size was small for both linacs; thus, more data need to be collected. From the sensitivity analysis of the BPR, for smaller *α*/*β* values, the BPR increases. The parameters differed for each tumor or normal tissue type. Moreover, in the LQ model, the effects of single high doses may be difficult to predict. The limitation of the current study is the lack of consensus regarding radiobiological formalism and cell parameters for different tumor and normal tissues. In the current study, the formalism and cell parameters were assumed to be fully validated, and a new framework for BPR calculation was suggested. Several models have been proposed for hypofractionation therapy schemes, such as the modified linear–quadratic, linear–quadratic–linear, and generalized linear–quadratic models.^18^, ^19^, ^20^ These biological models and various LQ parameters are applicable to the BPR model using the procedure developed in this study.

In addition to BPR, an EIR analysis was conducted to estimate the risk of economic cost in a medical institution. As shown by the results in Figs. 3 and 4, the patient and economic cost risks differ. Although the BPR for the minor interlock fault was the highest in Clinac iX and second highest in TrueBeam, the EIR effect was the least prominent. This interlock fault did not require part exchange, although the risk of biological effect was higher for the patient. Hence, the BPR may be reduced by confirming the alert and performing fault handling rapidly without considering the cost. The water temperature and quantity faults indicated BPRs and the smallest EIR in both linacs. A fault with a higher BPR and smaller EIR may exhibit the highest risk reduction impact if the user contacts an engineer immediately because the costs of labor and repair parts are low. Meanwhile, the EIR was higher for the monitor chamber and LaserGuard, though not of high frequencies and occurred only in TrueBeam. Our study showed that variations occurred in the DT ratio, cost of labor, and repair parts across machine types. These results are supported by a preceding study.^21^ Dufek et al. analyzed the DT of linacs at 13 institutions.^21^ They discovered variations in DT percentages across the institutions. Machine failures may depend on the introduction time of a machine or on an individual machine. Specifically, the EIR depends on the occurrence probability of machine failures and the costs of labor and repair parts, which vary according to machine type or vendor. Moreover, the tolerances of the machine fault differ for the two machines. Older machines may allow an operation with looser machine tolerances before a fault occurs. For example, unlike Clinac iX, TrueBeam has a collision interlock by LaserGuard. This might increase the risk for TrueBeam, which is a newer machine. From this discussion, it is clear that the machine failure risk faced by patients and institutions depends on many factors. Therefore, the manner in which failures occur in each institution must be understood such that a maintenance contract can be generated according to the risk level and the appropriate method of machine failure management can be determined.

Although scheduled preventative maintenance was performed in our institution by engineers, the interval of preventative maintenance was several months, which is insufficient. The preemptive maintenance model by the institution staff introduced in the previous study may resolve this problem.^22^ Able et al. attempted to create a model for the preemptive maintenance of medical linacs during MLC faults.^22^ They created a system that automatically and swiftly transferred log files and provided alerts regarding accumulation in the accelerator system.^22^ This system can improve the efficient deployment of service engineering resources, thereby resulting in fewer interruptions to treatment. Hence, preventive maintenance or remote support can reduce the DT, and the labor cost and biological effect can be reduced for planned after‐hours maintenance rather than for unscheduled maintenance.

Additionally, a backup linac can be used to reduce the risk if one is available during a machine failure. Xu et al. compared the passing rates of film measurement, ArcCHECK, and point doses for a clinical treatment plan among beam‐matched linacs.^23^ They demonstrated the availability of a swapping clinical plans among beam‐matched linacs. These back‐up systems can improve the throughput and reduce the BPR and EIR from machine problems. The sensitivity analysis of the EIR shows that using a backup machine can reduce the EIR. However, treatment with a backup machine poses another risk. Thus, a swift patient quality assurance for each treatment plan and a plan verification for the remaining MU and segment after a machine breaks down is necessary. Determination of the action level or threshold for the biological and economic risks by the machine faults is outside the scope of the current study. Biological and economic risks were independent. Therefore, the action level depends on the institution because the risk priority differs for each institution. The MFRA can be used to control and evaluate patient risk or economic risk. In future studies, the MFRA system will be expanded simulate risk reduction by performing MFRA after applying the risk‐reducing methods presented herein.

## 5 CONCLUSIONS

In machine failure, the risks faced by patients and institutions differ. The proposed MFRA contributes to the reduction in economic cost for institutions and biological effects on patients. Furthermore, the risk effects on patients and institutions differed between TrueBeam and Clinac iX. Identifying the machine failure risk faced by patients and institutions during treatment is critical for each institution and can offer prevention through model creation for preemptive maintenance to mitigate the risk, or through feedback to service engineering.

## CONFLICT OF INTEREST

The author have no other relevant conflict of interest to disclose.

## AUTHOR's CONTRIBUTIONS

DK, AS, and YO contributed to the design and implementation of the research and all authors contributed to the analysis of the results. DK wrote the first draft of the paper. All authors contributed to the drafting and editing of the manuscript and approved the final version.
